# Survey to Assess Knowledge and Reported Practices Regarding Blood Transfusion Among Cancer Physicians in Uganda

**DOI:** 10.1200/JGO.18.00143

**Published:** 2018-10-11

**Authors:** Henry Ddungu, Elizabeth M. Krantz, Warren Phipps, Sandra Naluzze, Jackson Orem, Noah Kiwanuka, Anna Wald, Isaac Kajja

**Affiliations:** **Henry Ddungu**, **Sandra Naluzze**, and **Jackson Orem**, Uganda Cancer Institute; **Noah Kiwanuka** and **Isaac Kajja**, Makerere University, Kampala, Uganda; **Elizabeth M. Krantz**, **Warren Phipps**, and **Anna Wald**, Fred Hutchinson Cancer Research Center; and **Warren Phipps** and **Anna Wald**, University of Washington, Seattle, WA.

## Abstract

**Purpose:**

Optimal decision making regarding blood transfusion for patients with cancer requires appropriate knowledge of transfusion medicine among physicians. We assessed blood transfusion knowledge, attitudes, and reported practices among physicians working at Uganda Cancer Institute (UCI).

**Materials and Methods:**

A cross-sectional self-administered survey of UCI physicians on their knowledge, attitudes, and practices regarding blood transfusion was conducted from June to September 2014. In consultation with transfusion medicine experts, 30 questions were developed, including 10 questions for each of the following three domains: knowledge, attitudes, and practices. For the knowledge domain, we created a knowledge score equal to the number of questions correctly answered out of 10.

**Results:**

Of 31 physicians approached, 90% participated. The mean knowledge score was 5.3 (median, 5.5), and 32% correctly answered at least seven of 10 questions. Almost all (96%) understood the importance of proper patient identification before transfusion and indicated identification error as the most common cause of fatal transfusion reactions. More than 60% of physicians acknowledged they lacked knowledge and needed training in transfusion medicine. Most physicians reported sometimes changing their mind about whether to provide a patient with a transfusion on the basis of opinion of colleagues and sometimes administering unnecessary transfusions because of influence from others.

**Conclusion:**

Although UCI physicians have some basic knowledge in transfusion, most reported gaps in their knowledge, and all expressed a need for additional education in the basics of blood transfusion. Transfusion training and evidence-based guidelines are needed to reduce inappropriate transfusions and improve patient care. Greater understanding of peer influence in transfusion decision making is required.

## INTRODUCTION

Transfusion therapy is indispensable in sub-Saharan Africa, where it is almost always administered as an emergency treatment of severe malarial anemia (in children), hemoglobinopathy, obstetric hemorrhage, and trauma.^1^ A study in Uganda to describe the use of blood at a tertiary care hospital found cancer to be the top indication for transfusion (33.5%), followed by pregnancy-related complications (12.4%) and sickle cell disease (6.9%).^2^ However, the high demand for transfusion does not meet the supply; for instance, the median whole-blood donation rate in sub-Saharan Africa is just 2.8 donations per 1,000 population, as compared with 36.4 donations per 1,000 population in high-income countries. Moreover, even when enough blood is donated, processing it into enough products needed for clinical use may not be possible because of financial and infrastructural inadequacies. In Uganda, for example, it costs approximately US$45.00 to US$50.00 to produce a unit of safe blood, and this cost becomes higher for platelets.

The clinical decision making behind whether to transfuse patients with cancer in resource-poor countries is poorly understood. Studies from other populations have shown that physicians’ lack of clinical knowledge and other nonclinical factors may influence their decision to transfuse.^3,4^ As a result, the tendency is inadvertently to transfuse inappropriately, with attendant risks and wastage of this rare resource. For instance, studies in Mwanza, Tanzania, demonstrated that 23% to 56% of blood transfusions were avoidable and that a major reduction in the number of blood transfusions could be achieved, particularly in the pediatric population.^5,6^ Improving patient care requires an understanding of factors that influence the decision to transfuse patients, which may guide the development of evidence-based guidelines and strategies for their implementation, with the latter involving change in physician behavior.^7^ The aim of this study was to assess the knowledge, attitudes, and reported practices of physicians at Uganda Cancer Institute (UCI) with regard to blood and platelet transfusions.

## MATERIALS AND METHODS

### Participants and Questionnaire

In consultation with transfusion medicine experts, we developed 30 questions, 10 for each of the three domains of knowledge, attitudes, and practices. We included topics considered essential for a clinician who is not a transfusion medicine specialist but whose practice includes regular ordering of blood products. We validated the questions by administering them to four internal medicine residents and two attending physicians, none of whom worked at UCI, to reveal inconsistent or confusing questions and then revised accordingly. We also consulted two transfusion medicine experts, one from the United States and the other from Canada, who had experience with transfusion in sub-Saharan Africa. These experts further reviewed the survey and determined the best or correct response to each question.

We used a convenience sampling method to select physician participants because of the small number of physicians at UCI at the time of the study. Our sample size included all physicians at UCI during the study period. All physicians, including residents, practicing at UCI from June to September 2014 were invited to complete a self-administered questionnaire on knowledge, attitudes, and practices regarding blood transfusion among patients with cancer. There was no specific time allocated in which to complete the questionnaire (participants were allowed to take it home with them), and the participants were encouraged to keep their responses confidential.

### Statistical Analysis

To assess physicians’ transfusion knowledge, we tabulated the responses for each of the 10 questions and computed a knowledge score, defined as the total number of questions answered correctly, for each physician. We used a histogram to show the distribution of this score and summarized the score using mean, median, and range. Attitude and reported practices questions were also tabulated. Analysis was performed using STATA software (version 14.1; STATA, College Station, TX). The Makerere University School of Medicine and the Fred Hutchinson Cancer Research Center research ethics committees approved the study.

## RESULTS

Questionnaires were given to 31 (91%) of 34 physicians at UCI during the 4-month study period, and of the 31, 28 (90%) returned the completed survey. Of the three who did not take the survey, one was traveling out of the country and two declined to take the survey. We did not collect data on the baseline characteristics of the physician respondents, because their small number at UCI would make it easy for the identification of the individual participants, thus compromising their confidentiality. However, their average level of training was a Master’s degree with most having more than 5 years of practice.

### Knowledge

**Fig 1:**
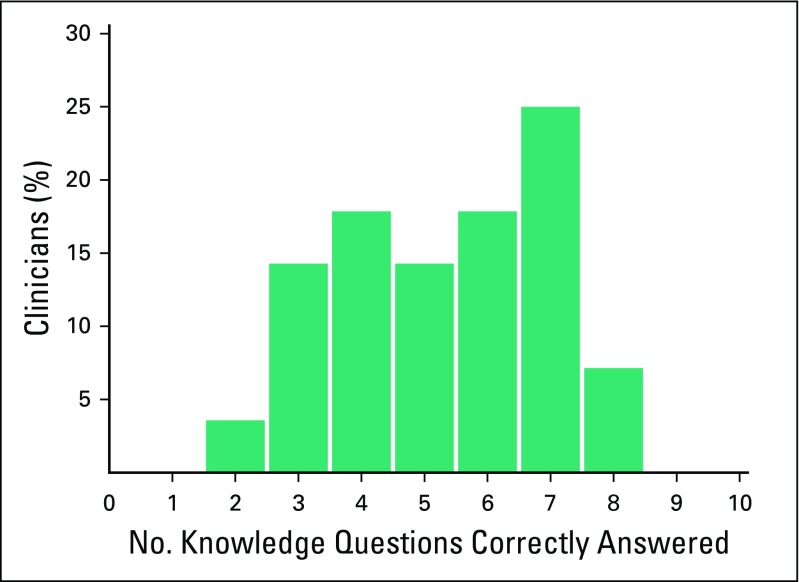
Distribution of number of knowledge questions correctly answered.

**Fig 2:**
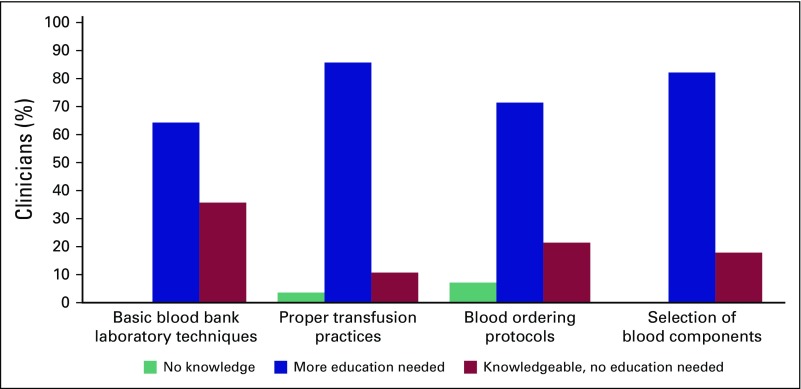
Physicians’ ranking of their knowledge on certain aspects of transfusion.

Among the 28 physicians, the mean knowledge score was 5.3, with a median score of 5.5 (range, 2 to 8); 32% of participants correctly answered at least seven of 10 questions (Table 1; Fig 1). Almost all responders (96%) understood the importance of proper patient identification before transfusion, and 64% correctly reported patient identification error as the most common cause of fatal transfusion reactions. Most physicians were knowledgeable about the practical aspects of administering blood transfusions. Eighteen physicians (64%) reported they would commence a transfusion right away after obtaining a blood unit on the ward. When given clinical vignettes describing specific transfusion scenarios physicians may encounter during their clinical work, 57% to 68% answered appropriately regarding the correct course of action (questions 7 and 9; Table 1). However, only 10 physicians (36%) knew the appropriate indications for transfusion of fresh frozen plasma. Fewer than half (42%) were knowledgeable about basic aspects of platelet transfusion (question 5; Table 1), and only 36% correctly identified bacteria as the most common transfusion-transmitted infection in Uganda. Furthermore, only two physicians (7%) had a clear understanding of transfusion-related acute lung injury. Physicians were also asked to rank their knowledge on various aspects of transfusion (Fig 2). For each item, more than 60% reported they either had little knowledge or needed more education in transfusion.

**Table 1:**
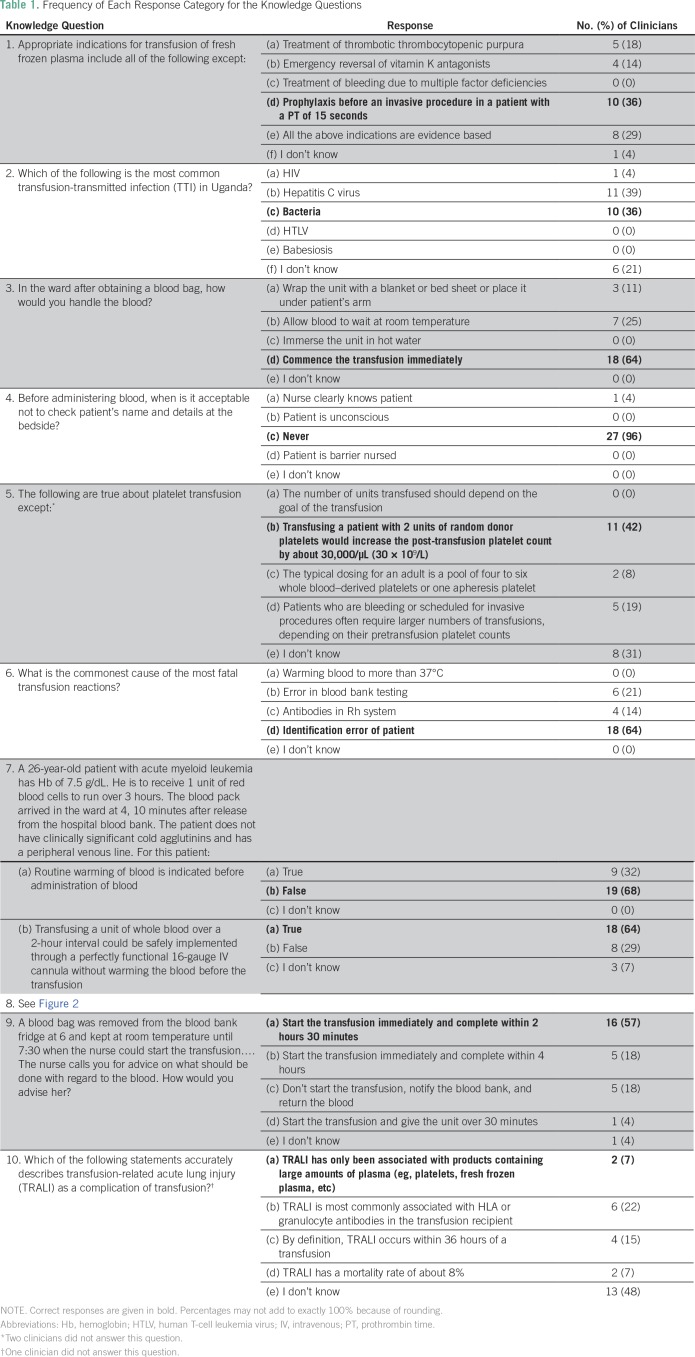
Frequency of Each Response Category for the Knowledge Questions

### Attitudes

Almost all participants (96%) strongly agreed that although donated blood was free, there were significant costs associated with blood processing and its administration (Table 2). Twenty-two participants (78%) agreed that they understood the risks and costs of allogeneic blood transfusion and that because of this they tried to minimize the use of blood components. Moreover, 90% acknowledged that in comparison with red blood cells, platelet transfusions were associated with a higher risk of transmission of diseases, and that they would use platelets with caution. When asked whether the Uganda Blood Transfusion Service should defer blood donations from people who had a clinical history of malaria within the past 3 years because there was no practical screening test for malaria, 27 participants (96%) disagreed.

**Table 2:**
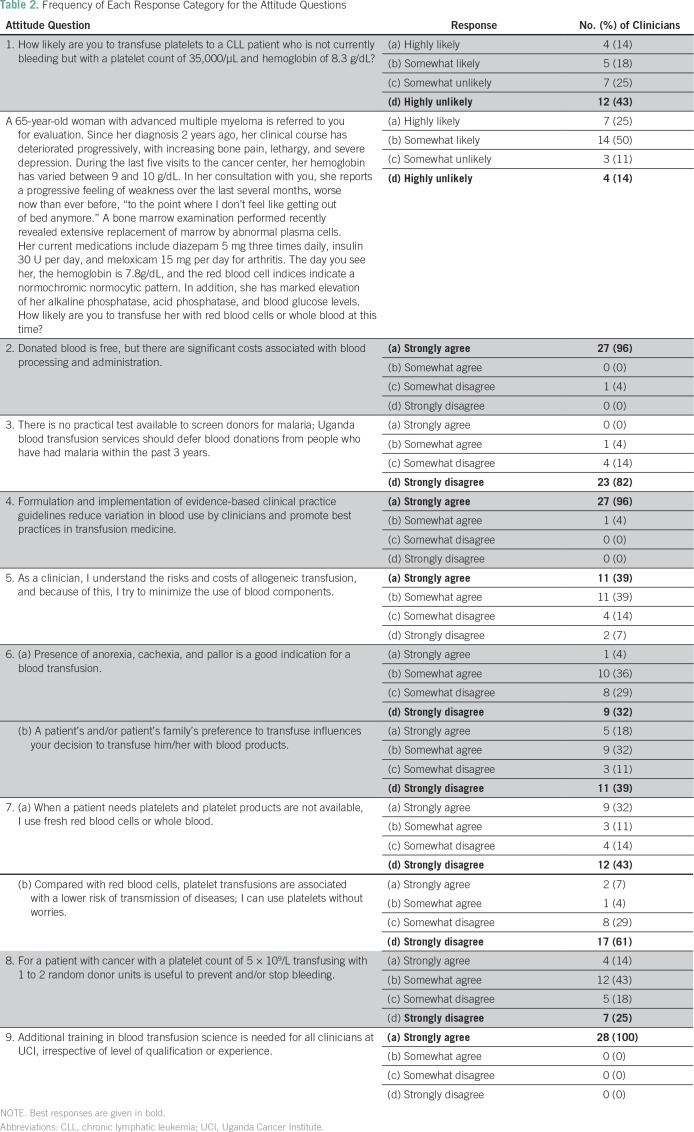
Frequency of Each Response Category for the Attitude Questions

Regarding the decision of when to transfuse, 61% of physicians reported they would not transfuse a patient on the basis of the patient’s symptoms of anorexia, cachexia, or pallor, but 50% would decide to transfuse because of a patient’s preference and/or that of his or her family. Attitudes regarding transfusion in specific clinical scenarios are summarized in Table 2 (questions 1, 2, and 9). All participants agreed that formulation and implementation of evidence-based clinical practice guidelines reduced variation in blood use by clinicians and promoted best practices in transfusion medicine. In addition, all respondents strongly agreed that additional training in blood transfusion science was needed for all clinicians, irrespective of level of qualification or experience.

### Transfusion Practices

Regarding their practices around transfusing patients with cancer (Table 3), all physicians reported routinely measuring hemoglobin before transfusing patients with red blood cells or whole blood, but most reported they would order a blood type (group) and cross match (71%) rather than a type and screen (25%) for patients with cancer admitted with anemia for whom no immediate transfusion was anticipated. Only seven physicians (25%) indicated they obtained signed consent from patients before administering a transfusion.

**Table 3:**
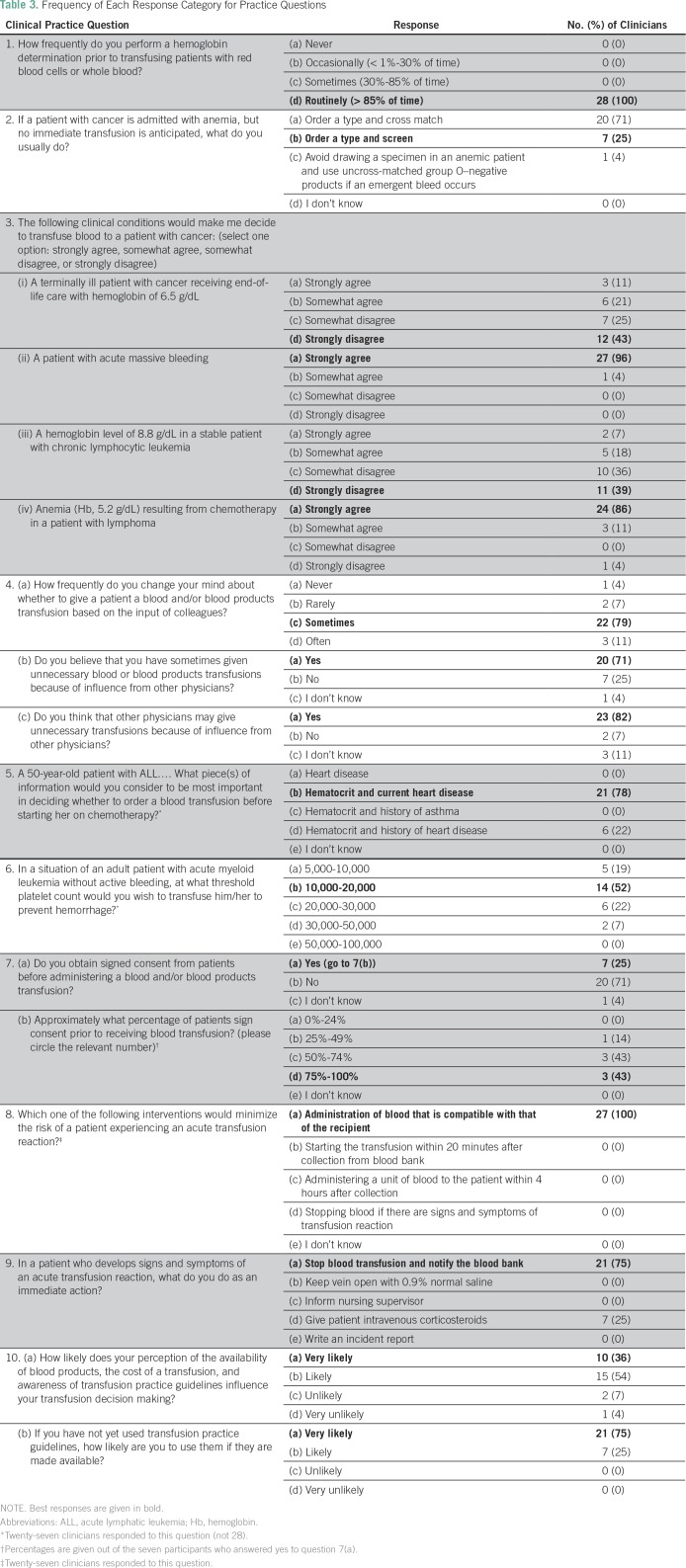
Frequency of Each Response Category for Practice Questions

Physicians were also asked whether certain clinical conditions would lead them to transfuse blood to a patient with cancer. Most (68%) responded that they would not transfuse a terminally ill patient with cancer receiving end-of-life care with a hemoglobin level of 6.5 g/dL or a stable patient with chronic lymphocytic leukemia and a hemoglobin level of 8.8 g/dL (75%). Conversely, all physicians indicated they would transfuse blood to a patient with cancer with acute massive bleeding, and all but one physician agreed that they would transfuse a patient with lymphoma and anemia (hemoglobin, 5.2 g/dL) resulting from chemotherapy. Physicians reported using a range of platelet count thresholds to determine whether to transfuse an adult patient with acute myeloid leukemia without active bleeding to prevent hemorrhage: five (19%) reported a threshold of 5 × 10^9^/L to 10 × 10^9^/L, 14 (52%) reported a threshold of 10 × 10^9^/L to 20 × 10^9^/L, six (22%) reported a threshold of 20 × 10^9^/L to 30 × 10^9^/L, and two (7%) reported a threshold of 30 × 10^9^/L to 50 × 10^9^/L.

The decision to transfuse patients with cancer was commonly influenced by others; physicians reported changing their mind about whether to administer a transfusion on the basis of the input of colleagues sometimes (79%) or often (11%). Twenty physicians (71%) believed that they had sometimes administered unnecessary blood or blood product transfusions because of influence of other physicians; 23 (82%) felt that this was also the case for other physicians. Fifteen physicians (54%) were likely and 10 (36%) very likely to have their transfusion decision making influenced by their perception of the availability of blood products, the cost of a transfusion, and their awareness of transfusion practice guidelines. When asked how likely they were to use transfusion practice guidelines if they were made available, all physicians answered they were likely or very likely to use such guidelines.

## DISCUSSION

In our survey, we found that UCI physicians have basic knowledge in transfusion, but they acknowledged gaps in their knowledge and expressed the need and desire for additional training. Most were fairly knowledgeable about the practical aspects of administering whole-blood and red cell transfusions but lacked knowledge about basic aspects of platelet transfusion. All physicians felt strongly there was a need for additional training in blood transfusion science. The physicians also felt that their decision to transfuse patients with cancer was commonly influenced by their colleagues and that such influence sometimes led to unnecessary blood transfusions.

The physicians’ knowledge score in this study was 5.3; only 32% of physicians correctly answered at least seven of 10 questions. This score (50.3%) was almost similar to that of a multicenter survey of internal medicine residents, where the mean score of correct responses was 45.7%.^8^ Two studies in Africa also found insufficient knowledge, but scores were higher than our finding, with 42.9% of medical staff in Mali^9^ and 50.8% of prescribers of blood products in Niamey, Niger,^10^ having good basic knowledge of transfusion. In Mozambique, another country with a limited supply of blood and one at high risk for transfusion-transmitted infections (TTIs), a survey among 216 health care providers on their perceptions and knowledge of blood transfusion found that providers were knowledgeable about transfusion but that some patient groups still received avoidable blood transfusions.^11^

In this study, physicians understood the importance of proper patient identification before transfusion and that patient identification error was a common cause of fatal transfusion reactions. This was also emphasized in the work performed by Maskens et al^12^ at Sunnybrook Health Sciences Centre in Canada, who found that errors resulting from inappropriate ordering of blood products and errors in sample labeling posed the greatest potential risk of patient harm.

Physicians were not familiar with TTIs, with only 36% correctly identifying bacteria as the most common TTI and 21% reporting they did not know the right answer. Importantly, transfusion-associated sepsis resulting from bacterial contamination is a frequent cause of mortality, representing 22% of 82 overall deaths related to transfusion in a French hemovigilance study.^13^ The highest bacterial contamination rate is observed with platelet concentrates (4.02 per 1,000 units), followed by red blood cells (1.71 per 1,000 units) and fresh frozen plasma (0.34 per 1,000 units). Potential interventions to reduce transfusion-associated bacterial sepsis include improvements to donor arm preparation, diversion of the first aliquot of whole blood, and introduction of bacterial testing,^14^ practices that are conducted in Uganda.

Malaria parasitemia is thought to be high among blood donors, especially those from highly endemic areas. A study in north-central Nigeria found a high percentage of apparently healthy blood donors harboring the malaria parasite.^15^ Although the WHO recommends that blood for transfusion be screened for TTIs, malaria screening is not performed in most malaria-endemic countries in sub-Saharan Africa, because there is currently no screening method that is practical, affordable, or suitably sensitive for use by blood banks in this region. In addition, implementation of any policy that advocates deferral of all such donors might have a significant negative impact on the availability of blood for transfusion.^16^ Indeed, in our study, almost all physicians (96%) disagreed with the idea of deferring blood donations from people who had malaria within the past 3 years. Of note, the clinical diagnosis of malaria is sometimes inaccurate, because most febrile illnesses are diagnosed as malaria in some areas, leading potentially to unnecessary discouragement or deferral of otherwise acceptable donors.

Treatment of anemia is important in palliative care, and blood transfusion is generally used for this purpose, although it is not clear if blood administered at the end of life is helpful.^17^ There are ethical questions about transfusion at the end of life, when decisions often involve seriously ill patients with evolving goals of care. Some argue that use of scarce resources such as blood must be balanced against maintaining adequate resources to treat future patients, thus putting the ethical principles of beneficence and social justice in conflict.^18,19^ Use of blood transfusion at the end of life may have an effect on survival of patients with cancer. A review of red blood cell transfusions in a palliative care unit in Adelaide, Australia, found that among patients, blood transfusions led to subjective improvement in a majority of recipients, although this correlated poorly with objective scale-based measures.^20^ Another study on the impact of blood transfusion on survival in patients with advanced cancer, in which anemic patients who had transfusion at admission were compared with those who were not transfused, found that patients who had blood transfusion at the end of life lived significantly longer than patients who were not transfused.^21^ In our study, most physicians indicated that they would not transfuse a terminally ill patient with cancer receiving end-of-life care on the basis of only a low hemoglobin level but would instead rely on a combination of other criteria and other prognostic indicators for survival. This is similar to findings in other studies, including a retrospective records review conducted in Italy.^22^

The decision to transfuse needs to be considered carefully, because exposure to transfused blood may be associated with risks, especially in patients with cancer. Avoidance of unnecessary exposure to blood components, particularly plasma and platelets, is preferable because of possible pathogen contamination. Unfortunately, because of limited transfusion knowledge, transfusions may be administered when not indicated, with a potential substantial risk to patients. Physicians in our study were often influenced by their colleagues in deciding when to transfuse, and in so doing, they believed that sometimes they could have administered unnecessary transfusions. A Canadian study to examine factors that guide blood transfusion decision making noted that both individual clinical appreciation and local unit (organizational) culture play a role in physicians’ decisions to transfuse patients.^23^ This suggests that promulgating appropriate guidelines would affect practice both by directly changing physician behavior and by changing the clinical norms in the medical community.

Our study is limited by small sample size; however, our participants represent the physicians caring for patients with cancer at the only national cancer referral hospital in Uganda. Our study is purely descriptive, and as such, our conclusions may not be as strong. Nonetheless, the findings, some of which corroborate studies from other countries, provide insight into cancer physicians’ knowledge and practices regarding transfusion in patients with cancer. Our findings may not be generalizable to other categories of health workers, because our study participants were from a fairly specialized group, but they may apply to physicians from other cancer treatment centers in sub-Saharan Africa.

Although some physicians had a good understanding of transfusion medicine, there is an imperative to improve transfusion knowledge, attitudes, and practices among UCI physicians. Indeed, most reported gaps in their knowledge and expressed a need for additional education in the basics of blood transfusion. Transfusion training and evidence-based guidelines to meet the unique situations faced by physicians in low- and middle-income countries would help to reduce inappropriate transfusions and improve patient care. Greater understanding of peer influence in transfusion decision making is required.

We recommend that medical training institutions in sub-Saharan Africa in general and Uganda in particular include transfusion in their curriculum to ensure physicians have knowledge of transfusion basics. There should also be in-service training for those clinicians already in practice on the basis of an accurate knowledge deficit assessment using a validated examination.^24^ To avoid unnecessary transfusions, guidelines need to be developed to help clinicians with the decision of whether to transfuse.
